# The first study on the effect of crocodile oil from *Crocodylus siamensis* on hepatic mitochondrial function for energy homeostasis in rats

**DOI:** 10.14202/vetworld.2022.986-997

**Published:** 2022-04-19

**Authors:** Kongphop Parunyakul, Krittika Srisuksai, Pitchaya Santativongchai, Urai Pongchairerk, Sumate Ampawong, Phitsanu Tulayakul, Wirasak Fungfuang

**Affiliations:** 1 Department of Zoology, Faculty of Science, Kasetsart University, Bangkok, Thailand; 2 Bio-Veterinary Sciences (International Program), Faculty of Veterinary Medicine, Kasetsart University, Bangkok, Thailand; 3 Department of Anatomy, Faculty of Veterinary Medicine, Kasetsart University, Bangkok, Thailand; 4 Department of Tropical Pathology, Faculty of Tropical Medicine, Mahidol University, Bangkok, Thailand; 5 Department of Veterinary Public Health, Faculty of Veterinary Medicine, Kasetsart University, Nakhon Pathom, Thailand

**Keywords:** crocodile oil, energy metabolism, liver, mitochondria, rat

## Abstract

**Background and Aim:**

Consumption of fatty acids (FA) can alter hepatic energy metabolism and mitochondrial function in the liver. Crocodile oil (CO) is rich in mono-and polyunsaturated FAs, which have natural anti-inflammatory and healing properties. In rat livers, we investigated the effect of CO on mitochondrial function for energy homeostasis.

**Materials and Methods:**

Twenty-one male Sprague-Dawley rats were divided into three groups at random. Group 1 rats were given sterile water (RO), Group 2 rats were given CO (3% v/w), and Group 3 rats were given palm oil (PO) (3% v/w). For 7 weeks, rats were given sterile water, CO, and PO orally. The researchers looked at body weight, food intake, liver weight, energy intake, blood lipid profiles, and mitochondria-targeted metabolites in the liver. The liver’s histopathology, mitochondrial architecture, and hydrolase domain containing 3 (HDHD3) protein expression in liver mitochondria were studied.

**Results:**

Body weight, liver weight, liver index, dietary energy intake, and serum lipid profiles were all unaffected by CO treatment. The CO group consumed significantly less food than the RO group. The CO group also had significantly higher levels of oxaloacetate and malate than the PO group. CO treatment significantly ameliorated hepatic steatosis, as evidenced by a greater decrease in the total surface area of lipid particles than PO treatment. CO administration preserved mitochondrial morphology in the liver by upregulating the energetic maintenance protein HDHD3. Furthermore, chemical-protein interactions revealed that HDHD3 was linked to the energy homeostatic pathway.

**Conclusion:**

CO may benefit liver function by preserving hepatic mitochondrial architecture and increasing energy metabolic activity.

## Introduction

The liver is a metabolic biotransformation hub and plays an important role in homeostasis. Among its many functions, the liver is in charge of physiological processes such as bile production, energy generation, as well as carbohydrate, proteins, and lipids metabolism [1]. Mitochondrial dysfunction is a broad term that encompasses changes in various metabolic pathways and mitochondrial component damage. Furthermore, mitochondrial dysfunction can lead to various negative outcomes, including oxidative stress, energy deficiency, accumulation of triglycerides (steatosis), and cell death [2]. Liver steatosis is caused by an increase in adipose lipolysis, which increases hepatic *de novo* lipogenesis, impairs the synthesis and/or secretion of very-low-density cholesterol, and causes triacylglycerol esterification dysfunction, or impaired mitochondrial β-oxidation [3]. Mitochondria are unique organelles that metabolize nutrients and are responsible for energy metabolism, free radicals generation, calcium homeostasis, cell survival, and cell death [4]. Metabolic activities are supported by hepatic mitochondria, which also contribute to the pathophysiology of insulin resistance and diabetes [5,6]. The mitochondria’s primary function is to synthesize adenosine triphosphate (ATP) through oxidative phosphorylation in accordance with metabolite oxidation through the tricarboxylic acid (TCA) cycle and β-oxidation of fatty acids (FA). A previous study found that a high-fat diet alters rat hepatocyte energy metabolism by inhibiting mitochondrial oxidative phosphorylation [7].

Over the last few decades, public concerns about the interaction of health and nutrition have grown rapidly. Fat is a necessary macronutrient in the human diet, and vegetable oils are now the most widely consumed fat in the world. Recently, attention has been given to the association between the fatty liver condition and the type of dietary fat consumed; the previous research has linked high-saturated FA (SFA) intake to the development of hepatic steatosis [8-10], a loss of whole-body fat oxidation [11-13], and ballooned cristae, as well as condensed matrix structures of hepatic mitochondria [14]. However, certain edible cooking oils, such as olive oil, soybean oil, and palm oil (PO), are consumed in greater quantities than other higher-quality oils. PO has higher ratios of SFA to mono-unsaturated FA (MUFA) and polyunsaturated FA (PUFA) than other cooking oils [15]. Previously, Li *et al*. [16] investigated the effects of a PO diet versus a low-fat diet on the expression of lipid breakdown-related genes and discovered that PO significantly reduced hepatic peroxisome proliferator-activated receptor alpha (PPAR-α) expression levels. When FAs inhibit PPARα, FA oxidation (FAO) and the resulting ATP production are reduced. Many previous studies have also linked SFA consumption to secondary diseases such as glucose intolerance, insulin resistance, dyslipidemia, cardiovascular disease, and hepatic steatosis [17-19]. Consumption of SFA-rich oils, such as PO, has been linked to the development of liver dyslipidemia, leading to mitochondrial dysfunction progression. Compared to other animal oils, crocodile oil (CO), extracted from the fatty tissues of *Crocodylus siamensis*, has a high concentration of MUFA and PUFA [20]. CO has been used for centuries by traditional practitioners and has been very effective in treating various ailments ranging from skin conditions to cancer [21]. CO has been used to treat skin rashes and promote wound healing in many previous studies [21,22]. Furthermore, a hamsters study found that a low MUFA and low PUFA to SFA ratio induced weight gain and body fat accumulation. In contrast, a high MUFA and high PUFA/SFA ratio prevented white adipose tissue accumulation [23]. It has been demonstrated that PUFA increases the expression of proteins involved in FAO while decreasing the expression of proteins involved in lipid synthesis [24].

However, the mechanism by which CO regulates hepatic mitochondrial-related energy metabolism in a rat model is largely unknown. We hypothesized that CO could be linked to changes in mitochondrial function in energy metabolic pathways in rat livers. As per our knowledge, this is the first report of the effect of CO on energy homeostasis via a hepatic mitochondrial function. The present study aimed to look into the effect of CO on hepatic energy metabolism and mitochondrial function in rats.

## Materials and Methods

### Ethical approval

The research conducted adhered to the Guidelines for the Care and Use of Laboratory Animals. The ethics committee of Kasetsart University Research and Development Institute, Kasetsart University, Thailand, approved this study (Approval No. ACKU61-VET-088).

### Study period and location

This study was conducted from October 2019 to May 2020 at the Department of Zoology, Faculty of Science, Kasetsart University, Bangkok, Thailand.

### Animal care, diets, and experimental design

In brief, Nomura Siam International Co. Ltd., Samutprakan province, Thailand, provided 21 Sprague-Dawley male rats (age: 7 weeks). This study used only male rats to prevent the effect of the estrous cycle on consumption behavior. The animals were individually housed in controlled environments (25±2°C on a 12-h light/12-h dark cycle). Throughout the study, rats had *ad libitum* access to food and water and were randomly assigned to one of three groups (n=7/group). Rats in Group 1 were given sterile water (RO), those in Group 2 were given CO (3% v/w), and those in Group 3 were given commercial PO (3% v/w) (PO). For 7 weeks, the animals were given sterile water, CO, and PO orally.

### CO preparation

When the meat was trimmed and prepared, CO was extracted using the wet cold-pressed oil method described by Santativongchai *et al*. [25]. Abdominal fat samples were obtained as a waste product from slaughtered *C. siamensis* (age: 3-5 years) collected from a crocodile farm in Nakhon Pathom Province, Thailand.

The samples were pressed through two layers of filter cloth with distilled water in a 1:1 (w/v) proportion. Following that, the solution was left undisturbed until the mixture separated. The upper clear oil fraction was then collected, evaporated, and stored at room temperature in a sealed container.

### Measurement of body weight, food intake, and energy intake of animals

Each rat’s food intake was measured by weighing the remaining chow daily between 11:00 and 11:30 h, and this was used to calculate energy intake. The daily energy intake per rat (kcal/day) was calculated as (food intake per rat*ME from standard chow) + ME from the treatment (ME is the total energy of the rat diets, which is 3.040 kcal/g in standard chow, 12 kcal in CO per day, and 13 kcal in PO per day). Throughout the experiment, body weight was measured every week.

### Sample collection

After the experiment, all animals were sacrificed with a lethal dose of pentobarbital sodium. Blood samples were drawn through cardiac puncture and centrifuged at 2200 *g* for 15 min at 4°C. The serum was kept at −20°C until further testing. The serum lipid profile was determined using a Hitachi 7080 analyzer (Hitachi, Tokyo, Japan) and included triglycerides, cholesterol, high-density lipoprotein, and low-density lipoprotein.

While performing a standard protocol for mitochondrial extraction, liver specimens were immediately collected, weighed to determine their index (organ weight/body weight), and stored in ice-cold homogenate buffer (0.32 M sucrose, 1 mM ethylenediaminetetraacetic acid, and 10 mM Tris-HCL; pH 7.4). Pieces of liver tissue were also separated for histopathology analysis and stored in a 10% neutral buffer formalin solution. Further liver samples were collected and homogenized in ice-cold phosphate-buffered saline (PBS) (20% w/v) before centrifugation at 2000 × *g* for 20 min at 4°C; the supernatants were stored at −80°C until further analysis.

### Mitochondrial energy-related intermediates analysis

The frozen supernatants were mixed with methanol in a ratio of 2:8 (v/v). After centrifugation (20,000 × *g* for 20 min at 4°C), the supernatants were evaporated in a −80°C freeze dryer. After that, the metabolites were redissolved in 500 μL of high-performance liquid chromatography (HPLC) buffer. HPLC analysis was performed on each 5 μL sample. Chromatography was carried out in the following manner: The injection volume was set to 5 μL and the column was kept at 40°C. An InertSustain C18 (150×4.6 mm) measuring 5 μm was used to separate the mobile phase, which contained 8% 1 N sulfuric acids. Gradient elution at a flow rate of 1 mL/min was used.

### Hepatic histopathology and fat accumulation analysis

Hepatocellular steatosis (macrovesicular and microvesicular) is a type of hepatotoxicity characterized by an abnormal accumulation of fat droplets in hepatocytes. Fixed livers were kept at room temperature (24-25°C) for 24 h in 10% formalin. To determine hepatocellular steatosis, the samples were embedded in paraffin, section, ed at 5-μm thickness, and stained with hematoxylin and eosin (H&E). Oil Red O (ORO) staining was used to determine the size and position of the fat droplets. Liver cryosections (5 μm thick) were fixed in PBS with 10% neutral-buffered formalin for 20 min, and then incubated in freshly prepared ORO solution for 10 min before being counterstained with hematoxylin for 20 s. A light microscope with a magnification of 200× was used to examine the sections. The number and total surface area of lipid particles were measured.

### Liver mitochondrial extraction

To avoid cellular damage, liver mitochondrial extraction was carried out within 1-2 h. Each group’s liver samples were pooled, weighed, homogenized, and washed in homogenate buffer. In a glass Potter-Elvehjem tissue grinder, liver specimens were homogenized with an appropriate volume of the homogenate buffer (4 mL homogenate buffer/1 g of the liver specimen). Several up and down strokes were performed with a motor-driven Teflon pestle at 600 rpm during this setup. The homogenates were then centrifuged at 1000 × *g* for 5 min at 4°C. The supernatants were collected and centrifuged at 15,000 × *g* for 2 min at 4°C. The mitochondrial pellets were collected and washed in homogenate buffer several times. The pellets were resuspended in an ice-cold final equilibrated buffer (250 mM sucrose, 5 mM KH_2_PO_4_, 10 mM Tris-HCl, and 2 mg/mL bovine serum albumin [BSA]; pH 7.2). Subsequently, 200 μL of the resuspended pellet was fixed in 2.5% glutaraldehyde in 0.1 M sucrose phosphate buffer for electron microscopy analysis. The protein content of mitochondria was determined using a spectrophotometer (NanoDrop-1000, Thermo Scientific, Wilmington, DE, USA) and a protein assay (Bio-Rad^®^, Hercules, CA, USA).

### Conventional electron microscopy

The ultrastructure of liver mitochondria was studied using electron microscopy. Each group’s liver specimens were fixed with 1% osmium tetroxide, dehydrated in graded ethanol, infiltrated in a series of LR white resin (EMS^®^, USA), embedded in pure LR white (EMS^®^), polymerized at 60°C for 48 h, cut into 100-nm-thick sections, and stained with lead citrate and uranyl acetate. A transmission electron microscope (model HT7700, Hitachi) was used to examine the ultrastructure of the liver. Hepatocytes with unimpaired or intact mitochondria were counted (50 cells per group) and compared with other treatment groups.

### Metabolite-protein and protein-protein interaction

We used STITCH v. 5.0 (European Molecular Biology Laboratory, Heidelberg, Germany) (http://stitch.embl.de/) to predict interactions between expected metabolites and proteins that interact with the targeted mitochondrial energy-maintenance proteins (haloacid dehalogenase-like hydrolase domain containing 3: HDHD3), such as the energy-related proteins (Prkaa1, Prkaa2, Prkab1, Prkab2, Prkag1, Prkag2, Akt1, Mtor, and Raptor) (Table-1) and mitochondrial energy-related metabolites (lactate, pyruvate, alpha-ketoglutarate, oxaloacetate, citrate, and malate). STITCH also provides a confidence score for each reported interaction, with values ranging from 0.40-0.70, 0.70-0.90, and 0.90-1.00, indicating medium, high, and highest confidence levels, respectively. Our study predicted the interaction with the required confidence thresholds (scores) of 0.40 or higher.

**Table 1:** Targeted energy metabolic-related protein list according to UniProt database.

Protein names	Gene names	Mass	Gene ontology (biological process)
5’-AMP-activated protein kinase catalytic subunit alpha-1 (AMPK subunit alpha-1)	Prkaa1	63,973	CAMKK-AMPK signaling cascade [GO: 0061762] Energy homeostasis [GO: 0097009] Fatty acid oxidation [GO: 0019395]
5’-AMP-activated protein kinase catalytic subunit alpha-2 (AMPK subunit alpha-2)	Prkaa2	62,258	Energy homeostasis [GO: 0097009]
5’-AMP-activated protein kinase subunit beta-1 (AMPK subunit beta-1)	Prkab1	30,394	Regulation of catalytic activity [GO: 0050790] Regulation of primary metabolic process [GO: 0080090]
5’-AMP-activated protein kinase subunit beta-2 (AMPK subunit beta-2)	Prkab2	30,227	Regulation of catalytic activity [GO: 0050790] Regulation of primary metabolic process [GO: 0080090]
5’-AMP-activated protein kinase subunit gamma-1 (AMPK subunit gamma-1)	Prkag1	37,386	Regulation of catalytic activity [GO: 0050790] Regulation of protein serine/threonine kinase activity [GO: 0071900]
Protein kinase AMP-activated non-catalytic subunit gamma 2	Prkag2	62,984	Regulation of catalytic activity [GO: 0050790] Regulation of fatty acid metabolic process [GO: 0019217]
RAC-alpha serine/threonine-protein kinase (Protein kinase B)	Akt1	55,735	Glucose homeostasis [GO: 0042593]; positive regulation of mitochondrial membrane potential [GO: 0010918]; protein kinase B signaling [GO: 0043491]
Serine/threonine-protein kinase mTOR (Mammalian target of rapamycin) (mTOR) (Mechanistic target of rapamycin) (Rapamycin target protein 1) (RAPT1)	Mtor, Raft1	288,794	Energy reserve metabolic process [GO: 0006112] Regulation of fatty acid beta-oxidation [GO: 0031998] Response to nutrient levels [GO: 0031667] TOR signaling [GO: 0031929]

### Immunogold labeling technique

The immunogold labeling technique was used to compare HDHD3 (mitochondrial energy marker) expression and localization of mitochondria between groups. The primary antibody marker (MyBioSource, USA) was rabbit polyclonal anti-HDHD3.

The mitochondrial pellet from the pooled liver extract in each group was secondary fixed, and tissue processing was carried out as previously described. For 30 min, the tissue sections were blocked with 50 mM glycine in PBS, followed by 5% BSA (EMS^®^) in PBS. They were then incubated for 1 h with 1:50 diluted primary antibodies before being treated with goat anti-rabbit immunoglobulin G conjugated with 10-nm gold particles (EMS^®^). Sections were washed several times with 0.1% BSA in PBS between steps. After rigorously washing the tissue sections with distilled water, a silver enhancement kit (Aurion R-Gent SE-EM kit; EMS^®^) was used to improve the contrast of the gold particle labeling. Finally, the sections were stained with lead citrate and uranyl acetate before transmission electron microscopy. The number of labeled gold particles was counted to determine the intact stage of liver mitochondria (50 mitochondria/group was evaluated).

### Statistical analysis

To better understand the biological processes, the targeted energy-related proteins (Prkaa1, Prkaa2, Prkab1, Prkab2, Prkag1, Prkag2, Akt1, Mtor, and Raptor) were identified using the NCBI and UniProt databases.

The data are presented as mean±standard error of the mean. Statistical analysis was performed by one-way analysis of variance followed by Tukey’s *post hoc* test in the R project statistical computing package (R core team, 2019). p<0.05 was considered statistically significant.

## Results

### Effect of dietary CO on final body weight, body weight gain, liver index, food intake, and energy intake

As shown in Table-2, CO- and PO-treated rats consumed significantly less food (15.84±0.28 and 14.98±0.31 g/day, respectively) than the RO group (18.82±0.25 g/day). However, there were no significant differences between groups in terms of final body weight, body weight gain, liver index, or energy intake.

**Table 2:** Effect of dietary crocodile oil on final body weight, body weight gain, liver weight, liver index, average food intake, and average energy intake of rats for 7 weeks.

Group	Final body weight (g)	Body weight gain (g)	Liver weight (g)	Liver index	Food intake (g/day)	Energy intake (kcal/day)
RO	522.57±19.25	26.17±2.67	12.45±0.69	0.024±0.00058	18.82±0.25^a^	57.21±0.78
Crocodile oil	534.43±5.98	33.86±5.80	13.67±0.72	0.026±0.0013	15.84±0.28^b^	60.16±0.86
Palm oil	528.86±19.51	34.67±4.54	13.34±0.59	0.026±0.00075	14.98±0.31^b^	58.53±0.95

Data are expressed as the mean±standard error of the mean. Different letters indicate statistically significant differences between groups (p<0.05)

### Effect of dietary CO on blood lipid profiles and liver mitochondrial energy-related metabolites

**Figure-1:**
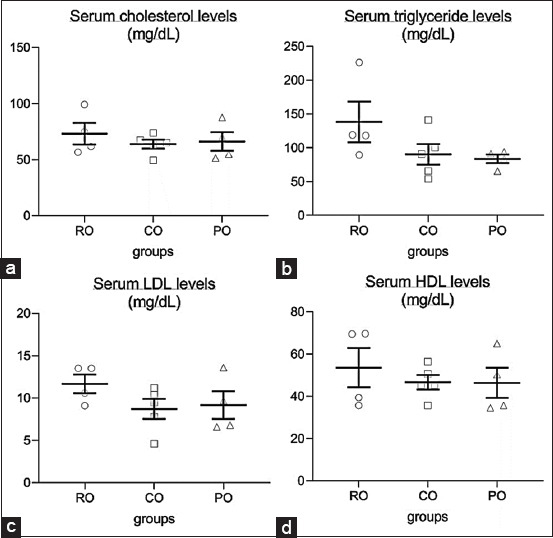
Effect of dietary crocodile oil on serum (a) cholesterol, (b) triglyceride, (c) low-density cholesterol, and (d) high-density cholesterol levels of rats for 7 weeks. Data are expressed as the mean±standard error of the mean.

Figure-1 depicts serum lipid profiles. The results revealed no statistically significant differences between the three groups. Interestingly, when compared to the RO group (138.25±30.14 mg/dL), both the CO (90.40±15.14 mg/dL) and PO (83.68±6.44 mg/dL) groups showed a decreasing trend in triglyceride levels.

**Figure-2:**
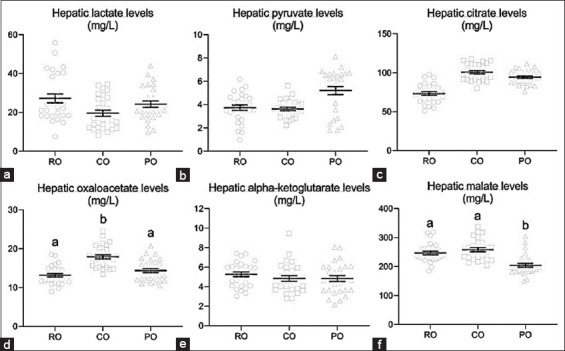
Effect of dietary crocodile oil on liver mitochondrial energy related-metabolites (a) lactate, (b) pyruvate, (c) citrate, (d) oxaloacetate, (e) alpha-ketoglutarate, and (f) malate levels. Data are expressed as the mean±standard error of the mean. Different letters indicate statistically significant differences between groups (p<0.05).

Figure-2 shows that the CO-treated group had significantly higher hepatic oxaloacetate levels (17.90±0.53 mg/L) than the RO- and PO-groups (13.17±0.45 and 14.37±0.53 mg/L, respectively) (Figure-2d). Meanwhile, there was no difference in hepatic malate levels between the CO and RO groups (246.92±6.02 and 258.03±6.72 mg/L, respectively), but the CO group had a significantly higher malate level than the PO group (204.41±6.61 mg/L) (Figure-2f). On the other hand, CO administration did not affect lactate, pyruvate, citrate, or alpha-ketoglutarate levels in the liver (Figure-2a-c, and e).

### Effect of dietary CO on hepatic lipid accumulation

**Figure-3:**
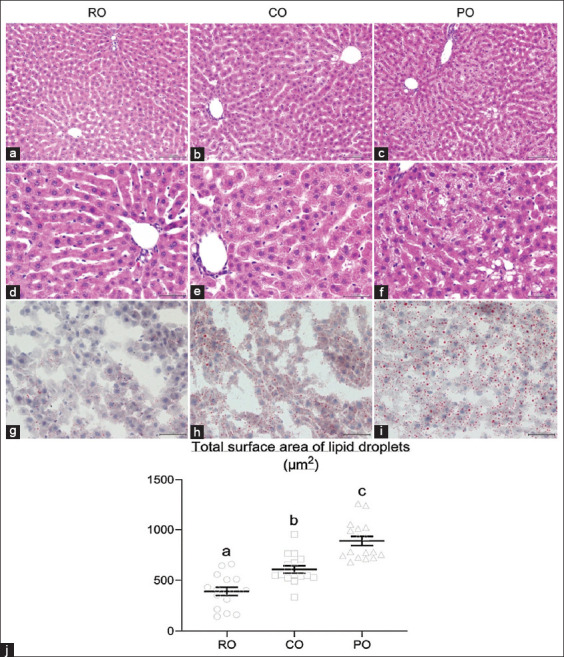
Histological analysis of liver lipid accumulation after 7 weeks of crocodile oil administration. (a-c) Hematoxylin and eosin (H&E) staining (200×), and (d-f) H&E staining (400×) with morphological forms of hepatic steatosis showing the hepatic steatosis in rat liver section; microvesicular steatosis was visible in crocodile oil (CO) group, whereas macrovesicular steatosis was visible in palm oil (PO) group. (g-i) Oil Red O staining showing the adipose deposition in rat liver section (400×); both oil-treated groups caused liver lipid deposits. PO showed more severe fat accumulation in the liver than CO group, and CO treatment exhibited more fat deposits than RO group. (j) The bar graph indicates the total surface area of the lipid droplets; the total surface area of fat droplets was significantly higher in the PO group than in the CO group. Data are represented by mean±standard error of the mean. Different letters indicate statistically significant differences between groups (p<0.05).

H&E staining was used to visualize hepatic steatosis in liver sections at 200× and 400× magnifications. Compared to the livers of RO-treated rats, liver histologic examination revealed microvesicular deposition (numerous small vesicles of fat in hepatocytes) in CO- and PO-treated rats. The size of fat droplets in CO, on the other hand, was clearly smaller than in PO. Furthermore, macrovesicular steatosis (a single large vacuole of fat filling up the hepatocyte) was found in the PO group, whereas the CO group showed had microvesicular steatosis (Figure-3a-f). ORO staining was used to examine the effect of CO supplementation on intracellular lipid levels in hepatocytes (Figure-3g-i). The number and size of hepatic fat droplets were lower in the RO group than in the CO and PO groups. Meanwhile, as shown in Figure-3j, the CO group had a significantly lower total surface area of lipid droplets in the liver than the PO group (608.40±35.67 and 889.23±44.77 μm^2^, respectively). The total lipid particle area seen in CO-treated rats, on the other hand, was significantly greater than that seen in RO-treated rats (391±40.67 μm^2^).

### Effect of CO on mitochondrial architecture and expression of HDHD3 in liver mitochondria

**Figure-4:**
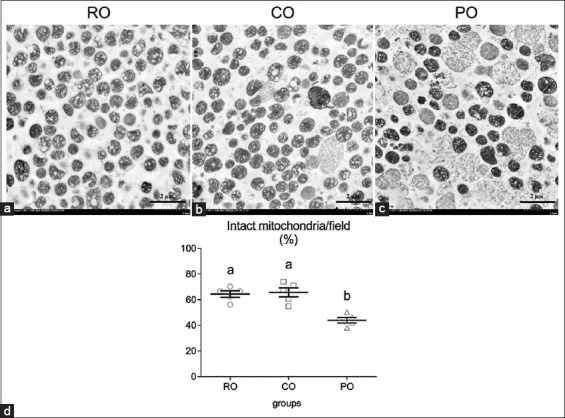
Comparison of electron micrographs of hepatic mitochondria conformation among the RO (a), crocodile oil (b), and palm oil (c) groups. The bar graph indicates the percentage of intact mitochondria (d), data are represented by mean±standard error of the mean. Different letters indicate statistically significant differences between groups (p<0.05). Scale bars=2 μm.

**Figure-5:**
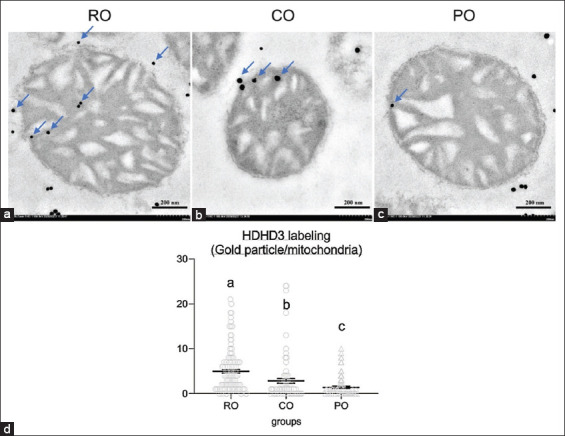
Comparison of the HDHD_3_ immunogold labeling at the intact stage of hepatic mitochondria among the RO (a), Crocodile oil (b), and palm oil (c) groups. The bar graph indicates the level of gold particles in HDHD3 labeling (d); data are represented by mean±standard error of the mean. Different letters indicate statistically significant differences between groups (p<0.05). Scale bars=200 nm. Blue arrows indicate a number of HDHD3-labeled gold particles.

After 7 weeks of CO administration, electron microscopy analysis was performed. The CO-treated rats had a similar percentage of intact mitochondria (65.71%) as the control RO group (64.45%), but significantly higher than the PO-treated rats (43.96%) (Figure-4). The expression of HDHD3, an energy-maintenance protein, was chosen to test their activity in the intact stage of mitochondria. The CO group had significantly higher levels of mitochondrial HDHD3 expression (2.86±0.52 gold particle/mitochondria) than the PO group (1.39±0.23 gold particle/mitochondria). Meanwhile, when compared with the CO and PO groups, the expression of HDHD3 protein was significantly higher in the RO group (4.98±0.40 gold particle/mitochondria) (Figure-5).

### The chemical-protein and protein-protein interaction analysis

**Figure-6:**
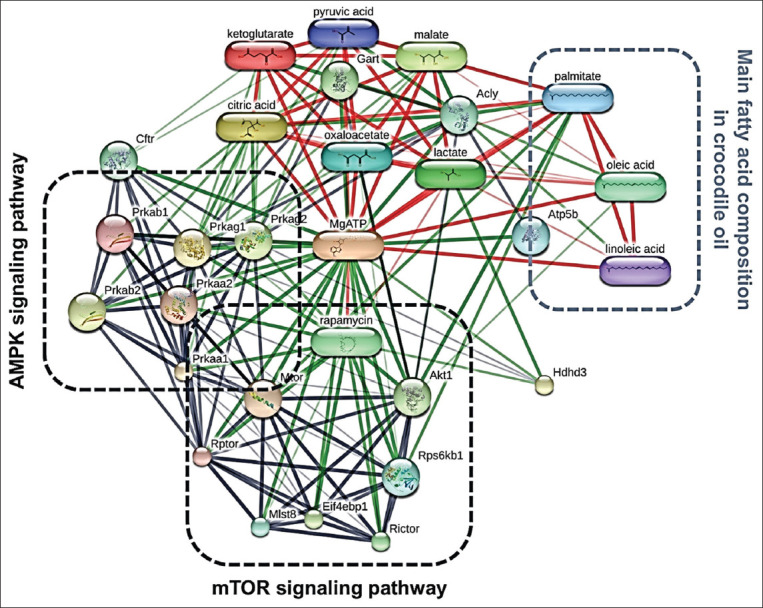
The chemical-protein and protein-protein interaction network of HDHD3 and the three main fatty acids of crocodile oil on the energy metabolic pathway in rat livers, as analyzed by STITCH v. 5.0.

The interaction network revealed that the HDHD3 protein was linked to the energy homeostatic pathway in the liver, as shown in Figure-6. HDHD3 interacted with chemicals and proteins in the mammalian target of rapamycin (mTOR) pathway (rapamycin; score=0.641), and the AMP-activated protein kinase (AMPK) signaling pathway (Prkag1; score=0.427, and Prkag2; score=0.469), both of which are energy regulatory pathways. Furthermore, HDHD3 interacted with energy-related metabolites (lactate; score=0.867) and was linked to ATP metabolic processes (ATP; score=0.696). In tabular form, the interaction score between HDHD3 and the energy-related target was shown (Supplementary Table-1). Meanwhile, the three most abundant FAs in CO (linoleic acid [LA], oleic acid [OA], and palmitic acid) interacted favorably with other chemicals involved in hepatic energy metabolism and proteins involved in the AMPK signaling pathway. As a result, CO can improve the activity of energy metabolism in the liver by upregulating the mitochondrial protein, HDHD3.

## Discussion

Our study is the first to look at the effect of CO on the changes in the health status of energy metabolism and mitochondrial function in the liver after 7 weeks of treatment. When compared to the PO group, CO could increase some key metabolites during mitochondrial energy metabolism, decrease hepatic steatosis, improve liver mitochondrial architecture, and upregulate energy-maintenance protein expression. These findings suggest that CO administration may be beneficial in the treatment of clinical liver metabolic damage. Meanwhile, there were no differences in final body weight, body weight gain, or energy intake between groups of CO-treated rats. We discovered that the primary MUFA and PUFA in CO were OA and LA, respectively. One of the essential FAs for humans and other mammals is PUFA. According to a previous study, PUFAs play an important role in the composition of all cell membranes, where they maintain homeostasis for proper membrane protein function and influence membrane fluidity, thereby regulating cell signaling processes, cellular functions, and gene expression [26]. In this regard, we agreed that CO consumption could have a protective effect on energy mitochondrial function.

First, our findings showed that an intake of dietary oil (CO and PO) was associated with lower food intake and higher fat accumulation in hepatocytes when compared to the RO group. A previous study looked at dietary FAs (SFA, MUFA, or PUFA) and found that they had a physiological effect on energy expenditure, energy intake, and body weight control [27]. The previous research on the effect of fat saturation on satiety and food intake reports that unsaturated FA-containing fats reduce food intake more than stearic acid-containing fats [28]. Furthermore, the current study found that PO, which is rich in SFA (palmitic acid), reduced food intake compared to the RO group. Many previous studies agree that SFA sources containing palmitic acid (coconut oil and palm kernel oil) provide greater satiety than other fat sources [29,30]. However, we propose no difference in food ingestion responses between types of dietary fat; this finding necessitates additional research into the mechanism by which saturation level can regulate food consumption and body weight management. On the other hand, the results showed that both types of dietary fat increased hepatic lipid accumulation compared to the RO group. It is widely accepted that dietary FA composition is an important determinant of hepatic lipid metabolism. In this context, a previous study found that increasing FAs in the diet, particularly SFA, played a significant role in the development of hepatic fat accumulation [31].

PO, which is rich in SFA like palmitic acid, increased fat accumulation in hepatocytes and induced mitochondrial dysmorphology in this study. The previous studies have linked abnormalities in mitochondrial morphology and function to fatty liver accumulation [32,33]. This point could contribute to the decrease in energy metabolism and mitochondrial function. PO is currently the most widely used edible oil in the food industry, although its relationship with liver energy metabolism remains unknown. Our current findings of H&E and ORO staining of lipid droplets in hepatocytes of rats fed PO revealed large lipid droplet size as macrovesicular steatosis and significantly increased surface area of intrahepatic triglycerides compared with the RO and CO groups, with CO exhibiting higher levels of liver content than the RO group. We propose that while the CO diet influences fat accumulation more than the normal rat diet, it may also prevent the development of lipid accumulation in the liver compared with the PO diet. The previous research has suggested that SFA may play a role in lipid metabolic toxicity, such as oxidative stress, and mitochondrial dysfunction [34]. PO altered hepatic metabolism and caused lipid accumulation via disturbed hepatocyte transcription, according to the previous study [35]. A subsequent study found that SFA sources containing palmitic acid can activate NOD-like receptor family pyrin containing 3 (NLRP3) inflammasomes, which are linked to the development of liver steatosis [36]. Meanwhile, Li *et al*. [16] investigated the effects of PO and a low-fat diet on the expression of lipid breakdown-related genes. The PO-treated group had significantly lower levels of expression of the PPAR-α (the gene involved in FAO), FA transport proteins, and their derivatives entering into the β-oxidation pathway to control energy homeostasis [37]. However, previous research has shown that olive oil, which is rich in MUFA, reduces liver fat accumulation by improving insulin resistance and increasing triglyceride release from the liver [38]. A subsequent study found that PUFA can reduce intracellular triglyceride deposition in the liver [39], and act as a potent inhibitor of hepatic lipogenesis [40]. As previously stated, a dietary CO, rich in MUFA (OA) and PUFA (LA), or PO, rich in SFA (palmitic acid) has a distinct effect on liver metabolism and hepatic fat accumulation because PO causes a significant increase in liver fat when compared to CO. PO, we believe, triggers energy metabolic deficiency by downregulating the transcription of energy homeostatic genes and causes liver damage by impairing mitochondrial function. It should come as no surprise that mitochondrial dysfunction plays a significant role in the development of excessive hepatic fat accumulation following PO administration.

Mitochondrial energy-related-metabolite levels reflect liver cell energy homeostasis. Our findings show that PO administration causes a decrease in some key energy-related metabolite levels. In contrast, CO administration causes a significant increase in oxaloacetate and malate levels in the TCA cycle compared to PO administration. The TCA cycle is the cell’s central metabolic hub and an important source of precursors for energy supply. However, the effects of CO on energy metabolism are not completely understood. Interestingly, according to a previous study, MUFA (OA) and PUFA (LA) are broken down in mitochondria to produce acetyl-CoA through FAO, a metabolite product that enters the TCA cycle to maintain ATP [41]. Furthermore, Liu *et al*. [42] investigated the mechanism by which omega-3 PUFA influences the TCA cycle in obesity; they discovered that PUFA may alleviate obesity by affecting mitochondrial function and restoring TCA cycle homeostasis, specifically the transcription and translation of TCA cycle enzymes such as citrate synthase, succinate dehydrogenase subunits A (SDHA), fumarate hydratase, and malate dehydrogenase 2 (MDH2) in HepG2 cells. Meanwhile, a recent study on the effect of SFA supplementation on the architecture and protein expression patterns of the murine heart discovered that protein expression patterns in animals fed a high-SFA diet show impairment of the TCA cycle and ATP synthesis [43]. Thus, PUFA-enriched diets could stimulate energy metabolic activity in rats by activating genes involved in the production of energy mitochondrial-related intermediates.

We observed the liver mitochondrial morphology and expression levels of proteins related to energy homeostasis to investigate the possible mechanisms of CO for the improvement of liver mitochondrial function. The current study’s findings revealed that PO had fewer healthy mitochondria than the CO and RO treatments. According to a previous study, a high-SFA diet caused hepatic fat accumulation which was consistent with impaired mitochondrial function and a dysregulated expression profile of mitochondrial dynamic proteins [44]. Furthermore, a high-SFA diet high in lard was linked to lower energy expenditure and the expression of genes controlling lipid metabolism and mitochondrial function [45]. Furthermore, the previous study in skeletal muscle cells has found that palmitic acid, the main SFA in PO, has a proclivity to induce mitochondrial DNA damage [46], as well as changes in mitochondrial morphology and function [47]. As a result, PO may contribute to mitochondrial form and function changes, leading to the development and progression of energy metabolic dysfunction in the liver. In contrast, according to our findings, CO-treated rats had a significantly higher percentage of intact mitochondria than the PO-treated group. Although there was little evidence of the effect of CO on mitochondrial function, studies on the effect of MUFA and PUFA on mitochondrial function were moderately published. According to a recent study on the effect of OA (the main MUFA of CO) on mitochondrial homeostasis at a cellular level, MUFA modulates mitochondrial function and lipid metabolism [48,49], resulting in increased mitochondrial ATP production. Furthermore, a previous study discovered that MUFA and PUFA inhibit the pro-inflammatory action of excess palmitic acid and counteract the morphological and functional changes in mitochondria [50]. Importantly, animals studies and a steatotic hepatocyte model support our hypothesis that PUFA improves mitochondrial morphology and has a beneficial effect on mitochondrial function recovery by regulating the mammalian target of the rapamycin complex 1 (mTORC1) pathway [51,52]. Previous research indicates that mTOR primarily acts through mTORC1, reducing oxidative capacity and altering mitochondrial morphology [53]. Many studies indicate that MUFA and PUFA intake have beneficial effects on mitochondrial dysmorphic features; these findings support our findings that CO restores liver energy metabolism by maintaining mitochondria ultrastructure and function.

Our current findings also confirmed CO’s protective effect on energy homeostasis; we discovered that CO not only maintains mitochondrial morphology but also increases energy production in the liver by upregulating mitochondrial HDHD3 protein. HDHD3 has been identified as a mitochondrial protein involved in cell energy maintenance [54]. The protein HDHD3 was found to be downregulated in PO-treated rats, whereas it was found to be upregulated in the CO-treated group. The interaction network between the HDHD3 and other predictable proteins involved in energy metabolism (Akt1, Prkaa1, Prkaa2, Prkab1, Prkab2, Prkag1, Prkag2, Mtor, and Raptor) were examined using the STITCH database (Figure-6). HDHD3 proteins were discovered to be associated with the protein kinases AMP-activated non-catalytic subunit gamma 1 (Prkag1), protein kinases AMP-activated non-catalytic subunit gamma 2 (Prkag2), and ATP production. Prkag1 and Prkag2 encode two isoforms of the AMPK g chain. AMPK is a key energy-sensing enzyme that monitors cellular energy status and has dual effects on mitochondrial function and structure [55]. Consuming PUFA-rich oil may increase hepatic AMPK activity and influence the regulation of hepatic lipid metabolism and gene expression [56].

Similarly, a previous study on the effects of krill oil (enriched with PUFA) on liver function found that the oil regulates genes and pathways involved in hepatic energy metabolism [57]. AMPK activation also causes mitochondrial biogenesis [58-60]. Mitochondrial biogenesis occurs in response to mitochondrial dysfunction in the liver to maintain cellular homeostasis against oxidative stress and injury by forming new mitochondria [61]. Surprisingly, our protein network result revealed a strong relationship between HDHD3, rapamycin, and an intermediate of mitochondrial energy metabolism. Rapamycin is an effective inhibitor of the mTOR protein kinase, which functions as a key integrator of nutrient signaling pathways [62]. mTOR promotes anabolic processes such as protein synthesis and is a key regulator of energy production in mitochondria [63], particularly TCA cycle intermediate production of mitochondrial metabolism. Furthermore, previous research found that inhibiting mTOR decreased levels of five key TCA cycle intermediates [63,64]. We believe that consuming PUFA-rich oil has a one-of-a-kind ability to improve energy metabolic associated mitochondrial structure and function. Finally, this research revealed that CO could improve liver energy homeostasis and mitochondrial contents by activating the mitochondrial HDHD3 protein.

## Conclusion

As per our knowledge, this is the first report of the effect of CO on energy homeostasis via a hepatic mitochondrial function. Our findings add to our understanding of the long-term effect of CO consumption on hepatic energy metabolism and mitochondrial function. Compared to the normal range, CO treatment reduced food intake and some key hepatic energy metabolite levels. It also slowed the progression of hepatic steatosis, improved mitochondrial morphology, and played an important role in energy metabolism maintenance by upregulating HDHD3 expression. Through the expression of protein-associated metabolic homeostasis, our findings may have clinical and therapeutic implications. CO has the potential to be a viable dietary fat substitute as well as a good choice for an economical therapeutic agent for treating metabolic energy disorders in the future.

## Authors’ Contributions

KP: Performed the study, analyzed the data, and wrote the manuscript. KS and PS: Performed the experiments. UP: Performed histological analysis. SA: Provided and analyzed mitochondrial function, and edited the manuscript. PT: Resource, conceptualization and revised the manuscript. WF: Conceived, designed, supervised the study and edited the manuscript. All authors read and approved the final manuscript.

## Supplementary Table

**Supplementary Table-1:** Confidence score of chemical-protein and protein-protein interaction between HDHD3 and energy-related targets according to STITCH database.

Node 1	Node 2	Node 1 accession	Node 2 accession	Confidence score
Hdhd3	Prkag1	ENSRNOP00000020380	ENSRNOP00000020093	0.427
Hdhd3	Prkag2	ENSRNOP00000020380	ENSRNOP00000062361	0.469
Hdhd3	rapamycin	ENSRNOP00000020380	5040	0.641
Hdhd3	MgATP	ENSRNOP00000020380	238	0.696
Hdhd3	lactate	ENSRNOP00000020380	612	0.867
